# ABO Discrepancy in Multiple Myeloma Initially Suspected as a Complication of COVID-19 Vaccination

**DOI:** 10.7759/cureus.109165

**Published:** 2026-05-19

**Authors:** Akanksha Agrawal, Tanvi Jha, Priyanka Gogoi, Preeti Diwaker

**Affiliations:** 1 Pathology, Shri Dharmasthala Manjunatheshwara (SDM) College of Medical Sciences and Hospital, Shri Dharmasthala Manjunatheshwara University, Dharwad, IND; 2 Pathology and Laboratory Medicine, Hamdard Institute of Medical Sciences and Research, Delhi, IND; 3 Transfusion Medicine, All India Institute of Medical Sciences, Guwahati, IND; 4 Pathology, University College of Medical Sciences and Guru Teg Bahadur (GTB) Hospital, Delhi, IND

**Keywords:** abo blood group, abo discrepancy, coomb’s test, covid 19 vaccination, plasma cell myeloma

## Abstract

The effect of COVID-19 infection and its vaccines on the immune system is still a subject of much research and debate. Caution is warranted to rule out known entities before considering these possibilities. We report a case of an incidentally detected ABO discrepancy initially suspected to be temporally associated with COVID-19 vaccination but diagnosed ultimately as multiple myeloma.

A 52-year-old male patient presented with weakness, easy fatigability, and chest pain following the second dose of the COVID-19 vaccine. His hemoglobin fell progressively, and he was advised blood transfusion. Blood grouping revealed an ABO discrepancy in forward and reverse grouping. The initial indirect Coombs test (ICT) showed 3+ reactivity, possibly due to non-specific interference from elevated serum proteins, but the repeat test was negative. Routine peripheral blood smear examination showed rouleaux formation and background blue-tinging. Saline replacement dispersed the RBC aggregates, confirming pseudo-agglutination. The patient also had a raised erythrocyte sedimentation rate (ESR). The patient was suspected of having a plasma cell dyscrasia, which was confirmed on serum electrophoresis, and this explained the ABO discrepancy.

M protein in plasma cell myeloma is known to cause group III ABO discrepancy due to rouleaux formation, which can be interpreted as pseudo-agglutination and can be a presenting feature. Though temporal association with vaccination may raise suspicion, a detailed work-up is necessary to avoid misattribution and missing underlying disease.

## Introduction

The impact of COVID-19 infection and vaccination on the immune system remains an area of ongoing research and debate. Unusual findings related to both COVID-19 infection and vaccination are increasingly being reported, with adverse events observed post-vaccination in some cases. Although vaccines have played a crucial role in controlling the spread of the virus, the occurrence of autoimmune diseases such as Guillain-Barré syndrome and the development of autoantibodies in recovered COVID-19 patients have raised concerns about potential immunological responses, possibly driven by molecular mimicry [1,2]. It is important to approach these cases with caution, ensuring known conditions are ruled out before attributing findings solely to COVID-19. Although literature does not provide information on the presence of ABO discrepancy due to the COVID-19 vaccine, it does explain inconsistency in blood grouping, especially in severe COVID-19 infection, which could be due to the temporary absence of antibodies leading to irregularities in blood group detection. This is explained on the basis that Anti-A or Anti-B act as viral neutralizing antibodies, which may disappear from the body during severe infections. Also, many studies have explained the preventive role of Anti-A antibodies in cases of COVID-19. Hence, A blood group is considered more susceptible to the infection [3,4]. Notably, elevated plasma globulin levels are seen in conditions such as multiple myeloma, Waldenström's macroglobulinemia, and Hodgkin's lymphoma. Type III ABO discrepancy occurs due to excess plasma proteins causing rouleaux formation and pseudo-agglutination. Plasma cell myeloma is a recognized cause. Approximately 60% of multiple myeloma patients present with anemia at diagnosis [5]. Here, we report a case of an incidentally detected ABO discrepancy initially linked to COVID-19 vaccination complications, but ultimately diagnosed as multiple myeloma.

This article was presented as a poster at the 62nd HAEMATOCON, November 2021.

## Case presentation

A 52-year-old male patient presented to the medicine outpatient department with symptoms of weakness, easy fatigability, and chest pain one week following the second dose of a COVID-19 vaccine, Covishield, Serum Institute of India (lot number not available). The patient experienced a progressive drop in hemoglobin levels, and a blood transfusion was recommended. Blood grouping tests revealed an ABO discrepancy between forward and reverse grouping. At 37°C, Anti-A: 4+, Anti-B: 0, Anti-D: 4+, A cell: mixed field (3+ and 1+), B cell: 4+. Similar discrepancies were observed at 25°C and 4°C with varying mixed field strengths (Table 1), indicating abnormal antibodies. The indirect Coombs test (ICT) of the patient using the tube method was positive (3+). This was likely due to non-specific interference from elevated serum proteins. A repeat ICT after further evaluation was negative.

**Table 1:** ABO and Rh reactions at various temperatures *Mixed field indicates dual population (DP) with graded agglutination strengths.

	A	B	Rh	A cell	B cell
37°C	4+	0	4+	3+/1+ (mixed field)*	4+
4°C	4+	0	4+	1+, 3+ (mixed field)*	4+
25°C	4+	0	4+	4+/1+ (mixed field)*	4+

On further testing with a three-cell panel, antibodies (4+) were found at 37°C for each panel, and an auto-control of 3+ was found at room temperature. These findings initially suggested an O blood group, but the presence of dual populations prompted further investigation.

Cross-matching with a low ionic strength solution (LISS) using the tube method was tried in six bags at 4°C; however, it was found to be incompatible with a dual population (DP) of 3+ in all the bags. Further, a 1+, 3+ DP grading of auto-compatibility was found at 4°C, hence representing the presence of abnormal antibodies. Cross-matching using the tube method was repeated with normal saline (NS) at 37°C with two bags, and the units were compatible.

**Figure 1:**
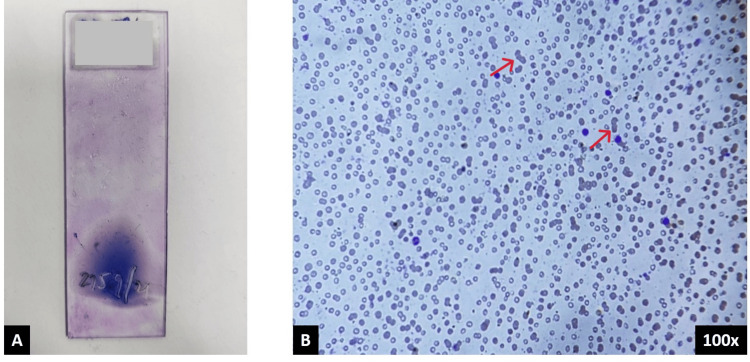
(A) Peripheral smear slide: blue hue of the peripheral smear. (B) Smear showing rouleaux formation (arrows), macrocytic cells, and bluish background; Leishman stain, 100x

The saline replacement technique was performed, which dissolved the agglutination, thus confirming rouleaux formation rather than true immune-mediated agglutination. Peripheral blood smear examination revealed rouleaux formation, background blue-tinging of the smear, macrocytic anemia, leukopenia, and thrombocytopenia (Figure 1).

**Figure 2:**
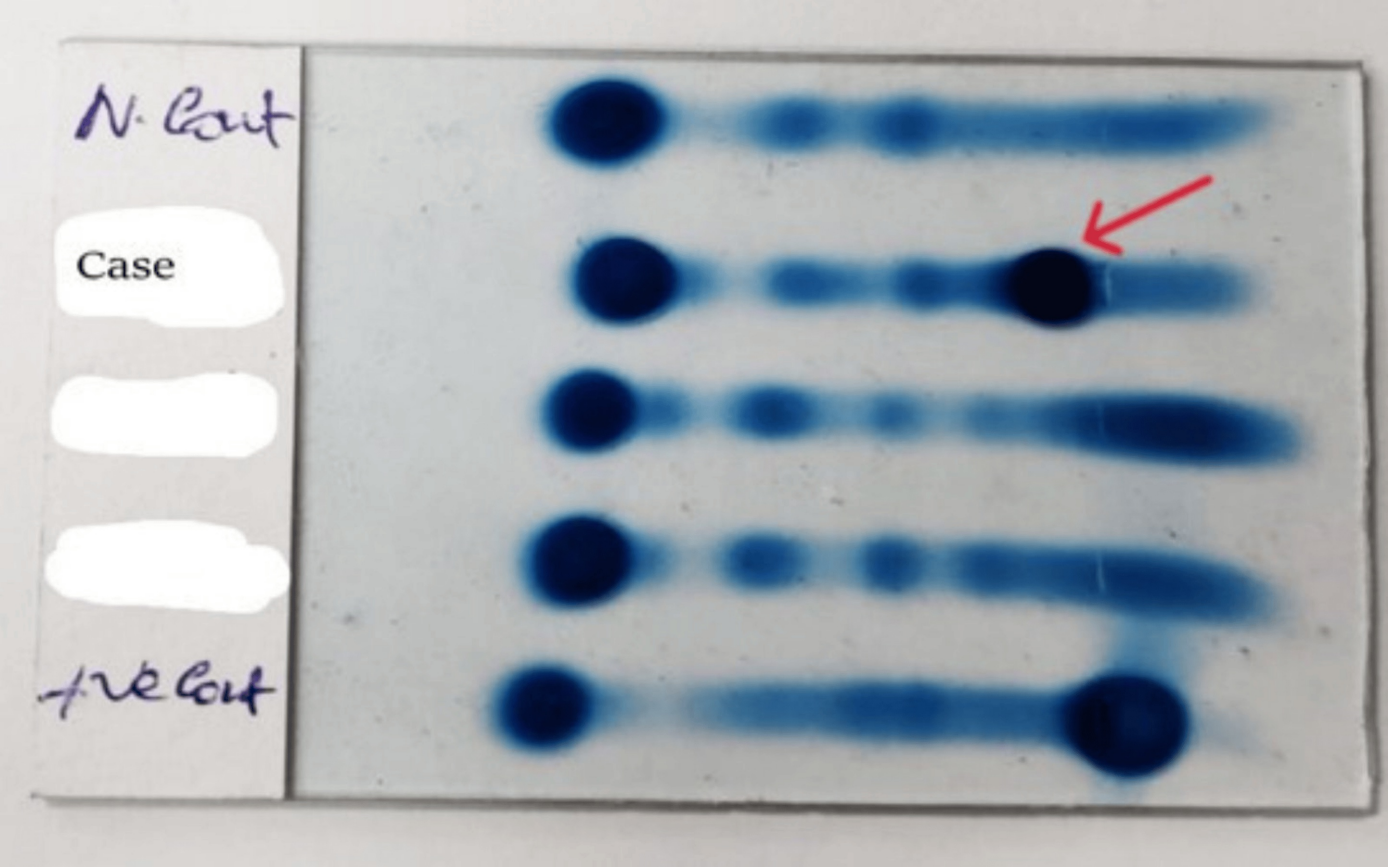
Serum electrophoresis showing a band at β-γ junction (M band) in the case sample (red arrow, row 2). Row 1 depicts negative control, and row 5 indicates positive control

The patient's erythrocyte sedimentation rate (ESR) was elevated, and both repeat direct Coombs test and ICT were negative. Based on these findings, the patient was suspected to have plasma cell dyscrasia. Serum electrophoresis confirmed the presence of monoclonal proteins (Figure 2), and immunofixation electrophoresis revealed elevated levels of total serum protein, serum globulins, IgG, and an abnormal kappa/lambda ratio. Total serum protein was 9.0 g/dL. The kappa/lambda ratio was 7.4. The patient was diagnosed with multiple myeloma, which explained the ABO blood group discrepancy.

## Discussion

Plasma cell dyscrasia encompasses a group of disorders characterized by the abnormal proliferation of plasma cells, the most common of which is multiple myeloma. These conditions often present with various systemic complications, including anemia, hypercalcemia, renal failure, and immunological anomalies [6]. One of the notable hematological findings is the alteration of plasma proteins, particularly immunoglobulins, which can contribute to abnormal blood test results, including ABO blood group discrepancies [5]. These discrepancies are especially common due to the production of M proteins, leading to pseudo-agglutination, which can be confused with immune-mediated reactions.

Post-COVID-19 vaccination complications have garnered significant attention, particularly due to reports of adverse immune responses following vaccination. While most reactions are mild and self-limiting, such as fever, pain, and fatigue, a few have raised concerns due to their severity or rarity. Guillain-Barré syndrome, myocarditis, and thrombocytopenia are some severe complications that have been linked to immune dysregulation post-vaccination. Given the immunological responses triggered by both COVID-19 infection and vaccination, it can be challenging to differentiate between immune responses due to vaccination and underlying conditions like plasma cell dyscrasia [1,2,7].

However, there is currently no strong literature stating that COVID-19 vaccination is a cause of ABO discrepancy. Most available work attributes these inconsistencies to the COVID-19 infection itself rather than its vaccination.

Multiple myeloma accounts for around 10% of all hematologic malignancies, with an incidence rate of 5-7 per 100,000 population annually. It predominantly affects older adults and is often underdiagnosed in its early stages due to vague symptoms like fatigue and back pain. The etiology of plasma cell dyscrasia is primarily genetic, though environmental factors such as chronic immune stimulation, exposure to ionizing radiation, and certain viral infections have been implicated. The malignant transformation of plasma cells leads to the overproduction of monoclonal immunoglobulins, which interfere with normal hematopoiesis and immune responses [8].

Complications from COVID-19 vaccination, while widely reported, remain rare relative to the total number of vaccinations administered globally. Most vaccination-related complications occur within the first few weeks and have mild to moderate symptoms. Severe immune-mediated reactions, such as Guillain-Barré syndrome, occur at an even lower incidence of approximately 1-2 cases per 100,000 doses [3,9].

Post-vaccination immune complications may arise from an exaggerated immune response, where molecular mimicry plays a role. SARS-CoV-2 antigens may cross-react with host antigens, leading to autoimmune phenomena. Molecular mimicry has been suggested as a cause of vaccine-related autoimmune disorders, where antigens in the vaccine trigger immune responses that cross-react with the body’s own tissues [10]. Vaccination in itself is not known to cause ABO discrepancy as per the literature available, and hence, to label ABO discrepancy in such a scenario, a thorough clinical and laboratory work-up must be done [2-4].

In plasma cell dyscrasia, the hallmark laboratory finding is the presence of monoclonal proteins (M proteins) in the serum and urine, typically detected through serum protein electrophoresis and immunofixation. A blood smear may show rouleaux formation due to increased serum protein levels. ABO discrepancies, as seen in this case, occur due to pseudo-agglutination caused by the elevated plasma proteins, particularly immunoglobulins [6]. The saline replacement technique is useful in differentiating pseudo-agglutination from true agglutination, as demonstrated.

Post-COVID-19 vaccination complications typically do not involve plasma protein abnormalities. However, in cases of immune-mediated conditions like autoimmune hemolytic anemia or thrombocytopenia, laboratory findings may include hemolysis markers (elevated lactate dehydrogenase, reticulocytosis) or thrombocytopenia. The absence of monoclonal proteins on electrophoresis is a crucial differentiating feature from plasma cell dyscrasia. Differentiating between post-COVID-19 vaccination complications and plasma cell dyscrasia is essential for accurate diagnosis and management. The presence of monoclonal proteins, rouleaux formation, and a significant increase in plasma globulins strongly favors a diagnosis of plasma cell dyscrasia over post-vaccination complications [2]. Peripheral smear examination and protein electrophoresis are invaluable diagnostic tools in these cases. Additionally, the presence of systemic features like bone pain, renal impairment, and anemia further supports plasma cell dyscrasia [6].

In contrast, vaccine complications may present with isolated immune phenomena without the characteristic laboratory findings of plasma cell dyscrasia. Clinicians should maintain a high index of suspicion for underlying conditions like multiple myeloma when presented with atypical post-vaccination findings, particularly in older adults with unexplained anemia or abnormal laboratory results [2,11].

Treatment for plasma cell dyscrasia focuses on controlling plasma cell proliferation and managing complications such as bone disease, anemia, and renal failure. Chemotherapy, proteasome inhibitors, immunomodulatory drugs, and monoclonal antibodies are commonly used to treat multiple myeloma. Blood transfusions, bisphosphonates, and dialysis may be required for symptomatic management [6].

In contrast, treatment for post-COVID-19 vaccination complications is usually supportive, focusing on managing the immune-mediated symptoms. Steroids, intravenous immunoglobulins, or plasmapheresis may be used in severe cases of autoimmune phenomena like Guillain-Barré syndrome or immune thrombocytopenia. Routine hematology investigations, particularly peripheral smear examination and serum protein electrophoresis, played a pivotal role in diagnosing this case of multiple myeloma, which was initially misinterpreted as a post-COVID-19 vaccination complication. Issuing blood in an ABO discrepancy involves a series of extensive cross-matching for antibodies, by which the most compatible blood is issued with a risk consent [1,7,12]. The early recognition of plasma cell dyscrasia prevented further mismanagement and allowed for the initiation of appropriate therapy. This case underscores the importance of considering underlying conditions when faced with atypical post-vaccination findings, especially in older patients with abnormal laboratory results. Accurate and timely diagnosis through detailed hematological work-up is essential to avoid misinterpretation and to provide optimal patient care.

## Conclusions

The presence of monoclonal proteins (M protein) in plasma cell myeloma can cause Type III ABO discrepancies due to rouleaux formation, mimicking agglutination and acting as an early disease indicator. In such cases, careful interpretation of blood grouping results is essential, and simple techniques such as saline replacement or microscopic examination can help differentiate rouleaux from true agglutination. Recognition of this discrepancy should prompt further evaluation, including serum protein studies, to identify an underlying plasma cell disorder at an early stage. Apparent temporal association with COVID-19 vaccination should be interpreted cautiously and should not delay appropriate diagnostic evaluation for underlying diseases such as multiple myeloma.
